# The Thermogenic Effect of Leptin Is Dependent on a Distinct Population of Prolactin-Releasing Peptide Neurons in the Dorsomedial Hypothalamus

**DOI:** 10.1016/j.cmet.2014.07.022

**Published:** 2014-10-07

**Authors:** Garron T. Dodd, Amy A. Worth, Nicolas Nunn, Aaron K. Korpal, David A. Bechtold, Margaret B. Allison, Martin G. Myers, Michael A. Statnick, Simon M. Luckman

**Affiliations:** 1 Faculty of Life Sciences, University of Manchester, Manchester M13 9PT, UK; 2 Division of Metabolism, Endocrinology and Diabetes, Department of Internal Medicine, University of Michigan, Ann Arbor, Ann Arbor, Michigan 48105, USA; 3 Lilly Research Laboratories, Eli Lilly and Company, Indianapolis, IN 46285, USA

## Abstract

Leptin is a critical regulator of metabolism, which acts on brain receptors (Lepr) to reduce energy intake and increase energy expenditure. Some of the cellular pathways mediating leptin’s anorectic actions are identified, but those mediating the thermogenic effects have proven more difficult to decipher. We define a population of neurons in the dorsomedial hypothalamic nucleus (DMH) containing the RFamide PrRP, which is activated by leptin. Disruption of Lepr selectively in these cells blocks thermogenic responses to leptin and causes obesity. A separate population of leptin-insensitive PrRP neurons in the brainstem is required, instead, for the satiating actions of the gut-derived hormone cholecystokinin (CCK). Global deletion of PrRP (in a *lox*STOP*lox*-*PrRP* mouse) results in obesity and attenuated responses to leptin and CCK. Cre-recombinase-mediated reactivation of *PrRP* in brainstem rescues the anorectic actions of CCK, but reactivation in the hypothalamus is required to re-establish the thermogenic effect of leptin.

## Highlights

• PrRP neurons in the hypothalamic dorsomedial nucleus are sensitive to leptin
• Cell-specific Lepr KO blocks leptin-induced thermogenesis and causes obesity
• Global deletion of PrRP attenuates responses to leptin and CCK
• Distinct PrRP populations are needed for thermogenesis and satiety effects

## Graphical Abstract

**Figure:**
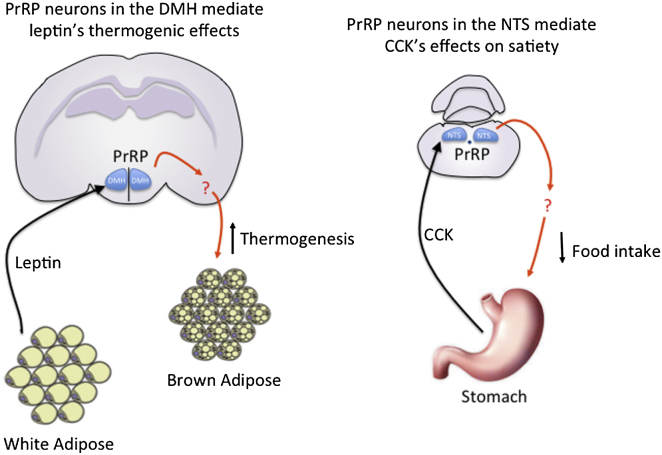

## Teaser

Leptin acts on the brain to regulate food intake and energy expenditure. Dodd et al. identify a population of PrRP neurons in the dorsomedial hypothalamus that directly respond to leptin to increase core body temperature. PrRP neurons in the brainstem are required, instead, for the satiating actions of gut-derived cholecystokinin.

## Introduction

Leptin is an adipokine, produced in proportion to white adipose tissue mass, which is critical for metabolic homeostasis. Deficiency in either leptin or its receptor, Lepr, leads to obesity due to increased feeding (Alingh Prins et al., 1986, McLaughlin and Baile, 1981) and reduced energy expenditure (through a decrease in core body temperature) (Trayhurn, 1979, Trayhurn et al., 1977). Leptin acts centrally to influence energy balance, since rescue of brain Lepr expression in otherwise Lepr-deficient mice reverses their obese and diabetic phenotype (de Luca et al., 2005). Substantial progress has been made in the identification of central cellular pathways involved in mediating the effects of leptin on energy intake. These include neurons of the arcuate hypothalamic nucleus, which contain either proopiomelanocortin (POMC) or neuropeptide Y (NPY)/agouti-related peptide (Balthasar et al., 2004, Mercer et al., 1996, van de Wall et al., 2007), as well as neurons in the ventromedial hypothalamic nucleus that contain SF-1/PACAP (Dhillon et al., 2006, Hawke et al., 2009). However, selective deletion of Lepr in first-order sensing neurons in the arcuate and ventromedial nuclei produces relatively mild obese phenotypes, suggesting additional populations of leptin-sensing neurons and, in particular, populations that mediate the important effects of leptin on adaptive thermogenesis and energy expenditure. It is hypothesized that Lepr-containing neurons of the dorsomedial hypothalamic nucleus (DMH) are an integral part of central thermogenic circuitry and important in leptin’s actions on energy expenditure (Enriori et al., 2011, Zhang et al., 2011), but the phenotypic identity of these neurons has not been confirmed.

We have proposed previously an important role for the RFamide prolactin-releasing peptide (PrRP) in energy homeostasis and, specifically, in brainstem pathways mediating the actions of the satiety factor CCK (Bechtold and Luckman, 2006, Lawrence et al., 2000, Lawrence et al., 2002). PrRP was originally described following deorphanization of the receptor GPR10 (Hinuma et al., 1998), but it was misnamed, as it has little or no physiological role in the control of prolactin (Dodd and Luckman, 2013, Samson et al., 1998). Genetic deficiency of either PrRP or GPR10 results in late-onset obesity and the loss of anorectic responses to CCK (Bechtold and Luckman, 2006, Gu et al., 2004, Takayanagi et al., 2008, Watanabe et al., 2005). Importantly, central injection of PrRP causes a reduction in food intake and increases in energy expenditure and core body temperature, which are additive with the effects of leptin (Ellacott et al., 2002, Lawrence et al., 2000, Lawrence et al., 2004). We have, thus, hypothesized that PrRP, in addition to mediating the satiating actions of CCK, is a critical target for leptin’s thermogenic signaling to the brain.

## Results

### The Expression of *PrRP* Is Regulated by Energy Status in Mice

**Figure 1:**
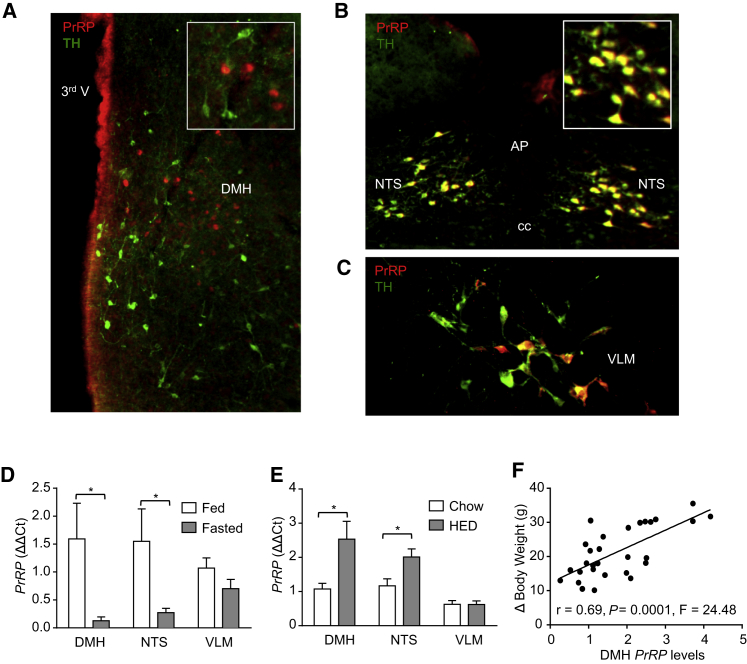
The Expression of *PrRP* in the DMH and NTS Is Regulated by Energy Status (A–E) Merged dual-label immunofluorescence for PrRP (red) and TH (green) in the (A) DMH, (B) NTS, and (C) VLM. Images reveal that PrRP neurons in the DMH do not coexpress TH, whereas 100% of PrRP neurons in the NTS and VLM do (dual-labeled cells display as yellow). Relative changes in *PrRP* mRNA in the DMH, NTS, and VLM following (D) a 24 hr fast or (E) 8 weeks on high-energy diet. (n = 6 per group, bars represent mean ± SEM; t test, ^∗^p < 0.05). (F) Linear regression analysis shows that the response of DMH *PrRP* mRNA to the diet is positively correlated with level of obesity in mice at 8 weeks (change in body weight).

PrRP is expressed in three distinct neuronal populations: one in the caudal DMH and two in separate regions of the brainstem—the nucleus of the tractus solitarius (NTS) and the ventrolateral medulla (VLM). As in the rat (Chen et al., 1999), the PrRP-expressing neurons in the mouse brainstem colocalize the catecholamine synthetic enzyme tyrosine hydroxylase (TH) (Figures 1A–1C) and correspond to subpopulations of the A2 and A1 noradrenergic cell groups, respectively. However, key to the studies described below, PrRP does not colocalize with TH in the DMH and is restricted to a small subregion posterior to the compact zone of this nucleus. In agreement with a proposed role in energy homeostasis, expression of *PrRP* mRNA in the caudal DMH and NTS is responsive to energy status. Specifically, laser capture microdissection and quantitative PCR demonstrated that *PrRP* expression is reduced in these nuclei (but not the VLM) in response to fasting and exhibits a complementary increase in expression in mice fed a high-energy diet for 8 weeks, an effect that was directly proportional to weight gained on the diet (Figures 1D–1F).

### PrRP Neurons in the DMH Mediate the Thermogenic Response to Leptin

**Figure 2:**
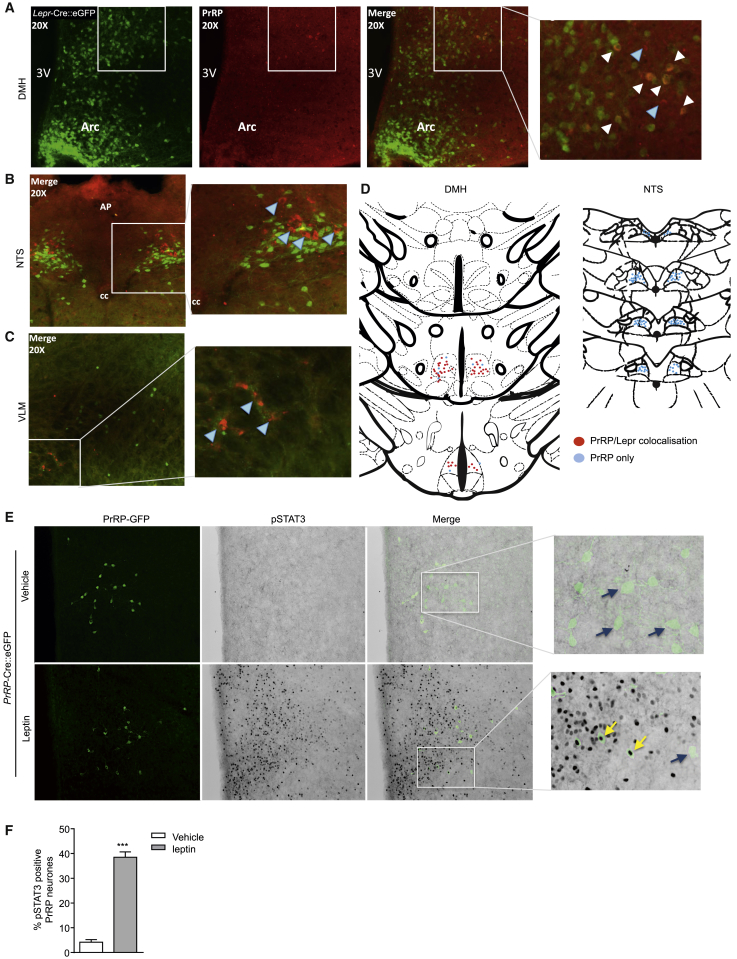
PrRP Neurons in the DMH Are Directly Responsive to Leptin (A–C) (A) Neurons in the DMH immunofluorescent for PrRP (red) in *Lepr*-Cre*::eGFP* (green) mice, and the merged image with digital zoom in adjacent panels. Representative merged images for (B) the NTS and (C) the VLM. PrRP and Lepr colocalize in cells of the DMH (merged yellow, indicated by white arrowheads) but not in the NTS or VLM. Blue arrowheads indicate cells expressing only PrRP. In the VLM, PrRP and Lepr neurons are not adjacent. 3V, third ventricle; AP, area postrema; Arc, arcuate nucleus; cc, central canal. (D) Anatomical maps showing the distribution of Lepr-containing PrRP neurons in the DMH and NTS. (E) Nuclear pSTAT3 induction in the DMH 60 min after vehicle or leptin (5 mg/kg, i.p) administration to *PrRP*-Cre::eGFP mice. PrRP neurons are immunostained for enhanced GFP. Black arrows indicate single stained PrRP neurons, and yellow arrows indicate PrRP neurons containing pSTAT3 immunoreactivity. (F) Bar graph showing that pSTAT3 is induced in at least 40% PrRP neurons in the caudal DMH in response to leptin administration (n = 6 per group, bars represent mean ± SEM; unpaired t test, ^∗∗∗∗^p < 0.0001). See also Figure S2, which shows that PrRP neurons in the NTS do not respond to leptin.

To determine if PrRP-containing neurons are direct targets for leptin, we used immunohistochemistry for PrRP on brain sections from *Lepr*-Cre*::eGFP* mice (a cross between a *Lepr*-IRES-cre recombinase mouse and a Rosa^eGFP^ reporter mouse, in which the Lepr-containing cells express enhanced green-fluorescent protein) (Leshan et al., 2009). Lepr is expressed extensively in the hypothalamus and can be seen to colocalize with PrRP neurons in the caudal DMH (Figures 2A and 2D). Lepr is also expressed in the dorsal vagal complex of the brainstem, including in the NTS, but is very sparse in the ventrolateral area of the medulla. There was no colocalization of Lepr and PrRP in either brainstem region (Figures 2B–2D), suggesting that any direct central effects of leptin are likely to be via the DMH population.

To explore leptin signaling via PrRP neurons, we generated a mouse in which the *PrRP* gene drives expression of IRES-Cre recombinase. Homologous recombination in embryonic stem cells was used to generate a line of heterozygous *PrRP*-Cre mice, which were then crossed with an eGFP (Z/EG) reporter mouse to produce *PrRP*-Cre::eGFP offspring. Targeted recombination is confirmed since eGFP in these mice is restricted exclusively to PrRP neurons (Figure S1 available online). As Lepr is linked to the STAT3 intracellular signaling pathway, the phosphorylated transcription factor (pSTAT3), which migrates to the cell nucleus upon activation, can be used as a measure of leptin signaling (Münzberg et al., 2003). A single, systemic injection of leptin (5 mg/kg body weight; intraperitoneal [i.p.]) causes a robust induction of pSTAT3 in *PrRP*-Cre::eGFP neurons in the DMH (Figures 2E and 2F) but none in *PrRP*-Cre::eGFP neurons in the NTS (Figure S2).

**Figure 3:**
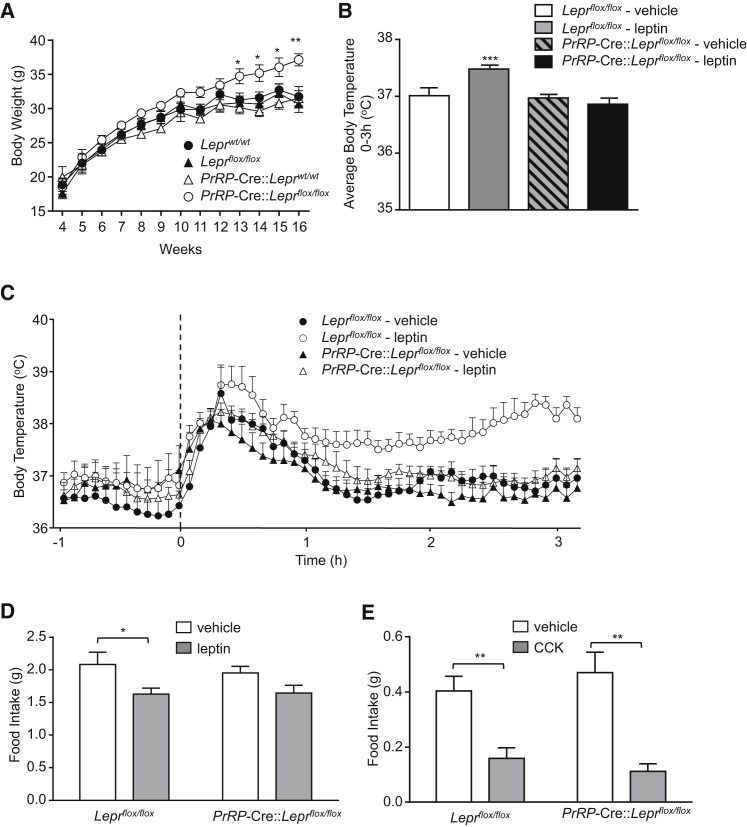
Lepr Receptors on PrRP Neurons in the DMH Mediate the Thermogenic Actions of Leptin *PrRP*-Cre mice were crossed with *Lepr*^flox/flox^ mice to knock out leptin receptors selectively in PrRP neurons. (A) Growth curves of *wt*, *Lepr*^flox/flox^, *PrRP*-Cre, and *PrRP-*Cre::*Lepr*^flox/flox^ male littermates. *PrRP-*Cre::*Lepr*^flox/flox^ mice, which lack leptin receptor expression in PrRP neurons, diverge in weight at 10 to 11 weeks of age (n = 8 per group, bars represent mean ± SEM; two-way ANOVA repeated-measures, ^∗^p < 0.05). (B and C) Leptin administration (5 mg/kg, i.p.) acutely increases body temperature in *Lepr*^flox/flox^ homozygotes and their (*wt*) littermates but not in mice lacking expression of leptin receptors on PrRP neurons (*PrRP*-Cre::*Lepr*^flox/flox^) (6 weeks old, n = 6 per group, bars represent mean ± SEM; two-way ANOVA, ^∗∗∗^p < 0.001). Dotted line in (C) represents time of injection. (D) Leptin reduces nocturnal food intake 4 hr after injection in *wt* mice; however, this effect is not significant in *PrRP*-Cre::*Lepr*^flox/flox^ mice (6 to 7 weeks old, n = 5 to 6, bars represent mean ± SEM; two-way ANOVA, ^∗^p < 0.05). (E) CCK (20 μg/kg, i.p.) causes a robust decrease in nocturnal food intake 1 hr after injection in *wt* and *PrRP*-Cre::*Lepr*^flox/flox^ mice (6 weeks old, n = 5 to 6 per group, bars represent mean ± SEM; two-way ANOVA, ^∗∗^p < 0.01).

We next utilized a transgenic “floxed” leptin receptor gene mouse (Lepr^flox/flox^) (McMinn et al., 2004) in order to selectively knock out Lepr only in cells expressing PrRP. Heterozygous *PrRP*-Cre mice were crossed with *Lepr*^flox/flox^ mice, and their offspring were mated to produce the homozygous littermates required for phenotyping experiments. Compared with wild-type (*wt*), single transgenic *PrRP*-Cre, and *Lepr*^flox/flox^ control littermates, the *PrRP*-Cre::*Lepr*^flox/flox^ mice display late-onset obesity (Figure 3A). The difference in body weight at 16 weeks of age (approximately +17%) is comparable with that of mice lacking Lepr in either POMC or SF-1 neurons (Dhillon et al., 2006) but is still significantly less than that reported for complete Lepr-deficient *db*/*db* mice (+60%) (Hummel et al., 1966). The obesity in *PrRP*-Cre::*Lepr*^flox/flox^ mice is not due to greater food intake, as there was no difference between littermates at any age, but is instead due to lower energy expenditure (Table S1). These mice have slightly lower average core body temperature over the 24 hr period, measured remotely in freely behaving mice by radiotelemetry (Table S1; a surrogate measure of adaptive thermogenesis and energy expenditure), as seen in complete leptin-receptor-deficient *db*/*db* mice (Trayhurn, 1979). Furthermore, the action of leptin to increase core body temperature is completely blocked in preobese (6 weeks of age), *PrRP*-Cre::*Lepr*^flox/flox^ mice (Figure 3B and 3C). As leptin is thought to activate sympathetic output to brown adipose tissue, we measured gene expression for uncoupling protein, UCP-1. An approximate 2-fold increase in *Ucp*-1 mRNA, following injection of leptin, is blocked in *PrRP*-Cre::*Lepr*^flox/flox^ mice (Figure S3A). Importantly, although the reduction in night-time feeding after leptin is slightly attenuated in *PrRP*-Cre::*Lepr*^flox/flox^ mice, these mice still respond normally to the satiating effect of CCK (20 μg/kg, i.p.) (Figures 3D and 3E). The dependence of central Lepr signaling for the metabolic actions of leptin is well established (de Luca et al., 2005), so it is important to demonstrate that Lepr signaling is not generally compromised in the *PrRP*-Cre::*Lepr*^flox/flox^ mice. Thus, using quantitative PCR, we demonstrated normal expression of Lepr in different tissues (data not shown). Furthermore, we show that, like their littermates, *PrRP*-Cre::*Lepr*^flox/flox^ mice respond to leptin with a robust induction of pSTAT3 generally in the hypothalamus (Figure S3B). Our results strongly implicate an integral role for PrRP neurons, and specifically those in the DMH, in mediating the effects of leptin on thermogenesis.

### Genetic Ablation of *PrRP* Results in Obesity

**Figure 4:**
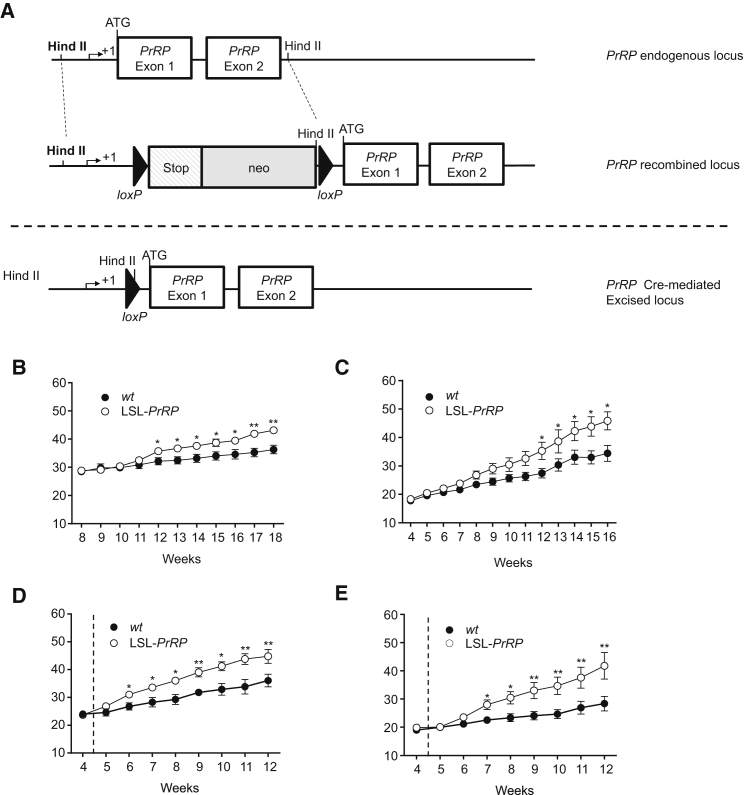
Transgenic Mice Expressing a Transcriptionally Silenced *PrRP* (LSL-*PrRP*) Allele Are Obese (A–E) (A) A *PrRP* inducible knock-in allele was generated by inserting a loxP-flanked stop codon between the transcription initiation (+1) and the ATG of the *PrRP* coding sequence. Tissue-specific coexpression of Cre-recombinase will remove the stop codon and rescue *PrRP* transcription. Growth curves of *wt* and LSL-*PrRP* (B) males and (C) females when fed standard chow. Body weights were significantly higher in the LSL-*PrRP* animals when compared with *wt* littermates at 12 weeks of age (see Table S1 for biometric data at culling and Figure S4 for validation of lack of PrRP expression). Growth curves of *wt* and LSL-*PrRP* (D) males and (E) females when fed a high-energy diet. Dotted line represents transition from standard chow to high-energy diet. Body weights were significantly higher in the LSL-*PrRP* animals, when compared with *wt* littermates, 2 to 3 weeks later (n = 6 per group, bars represent mean ± SEM; two-way ANOVA repeated measures; ^∗^p < 0.05; ^∗∗^p < 0.01).

The studies above demonstrate the essential role of PrRP-expressing neurons in the thermogenic effects of leptin. We next set out to demonstrate that PrRP signaling itself is central to the leptin pathway mediating thermogenesis. For this, we generated transgenic mice containing a *lox*STOP*lox* (LSL) codon upstream of the *PrRP* gene (Figure 4A). First, heterozygous LSL-*PrRP* mice were crossed to produce homozygous wild-type and LSL-*PrRP* littermates for phenotype comparisons. The lack of PrRP expression in homozygous LSL-*PrRP* mice was confirmed by both immunohistochemistry and by relative quantitative PCR (Figure S4). The body weights of both male and female homozygous LSL-*PrRP* mice, which lack expression of PrRP throughout the body, diverge from congenic littermates at 8 to 9 weeks of age, and become significantly obese at approximately 12 weeks (Figures 4B and 4C). By 18 weeks of age, the mice exhibit significantly increased adiposity (without a difference in somatic growth) and are hyperleptinaemic, hyperinsulinaemic, and hyperglycaemic (Table S1). Interestingly, in this model, the obese phenotype appears to be predominantly due to greater energy intake, as no decrease in energy expenditure was measured (Table S1). This is similar to the phenotype of *PrRP* knock-out mice generated previously, but which do not diverge in weight significantly until 18 weeks on normal chow (Takayanagi et al., 2008). LSL-*PrRP* mice are also sensitive to diet-induced obesity, when maintained on a high-energy diet (60% energy as fat) from 4 weeks of age (Figures 4D and 4E). Here, as with *PrRP* knock-out mice, their body weights diverge significantly from wild-type littermates within 2 weeks of the change in diet.

**Figure 5:**
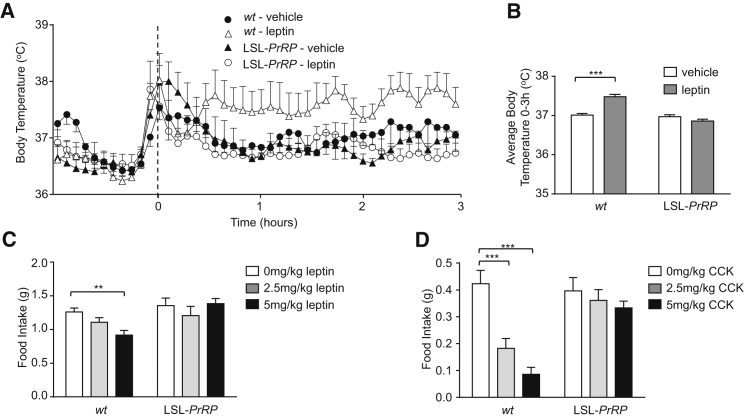
LSL-*PrRP* Mice Are Resistant to Leptin’s Thermogenic Actions (A and B) Preobese mice lacking *PrRP* (LSL-*PrRP*) do not respond to leptin with an increase in body temperature (6 weeks old, n = 8 per group, bars represent mean ± SEM; two-way ANOVA, ^∗∗∗^p < 0.001). (C) Leptin significantly decreases nocturnal food intake in *wt* mice, but this effect is abolished in LSL-*PrRP* mice (6 to 7 weeks old, 2 hr food intake data; n = 6 per group, bars represent mean ± SEM; one-way ANOVA, ^∗∗^p < 0.01). (D) CCK causes a dose-dependent decrease in nocturnal food intake in *wt* mice, but this effect is abolished in LSL-*PrRP* mice (6 weeks old, 1 hr food intake data; n = 6 per group, bars represent mean ± SEM; one-way ANOVA, ^∗∗∗^p < 0.001).

Preobese (6 weeks of age), LSL-*PrRP* mice maintained on normal chow were tested for their responses to both leptin and CCK. LSL-*PrRP* mice fail to increase core body temperature in response to systemic leptin administration (Figures 5A and 5B) or to induce brown adipose *Ucp*-1 (data not shown), supporting our view that PrRP mediates leptin’s thermogenic effect, though not pinpointing the source of PrRP in this role. The injection of either leptin or CCK causes a dose-dependent decrease in normal, night-time food intake in *wt* littermates (Figures 5C and 5D). By contrast, LSL-*PrRP* mice display no significant effects of either leptin or CCK on food intake.

### Selective Rescue of Brain *PrRP* Identifies Key Role of Hypothalamic Neurons

Having used the LSL-*PrRP* mouse to demonstrate that PrRP is critical for leptin-induced thermogenesis, we then used Cre-recombinase technology to reactivate *PrRP* expression, since crossing LSL-*PrRP* mice with mice possessing tissue-specific Cre leads to excision of the upstream STOP codon at *lox*P sites (Figure 4A). As *PrRP* is expressed in peripheral tissues (the pituitary and adrenal glands) that could have important metabolic consequences, we first crossed LSL-*PrRP* mice with *nestin*-Cre mice in order to rescue expression of *PrRP* only in the brain. First, we demonstrated that LSL-*PrRP* mice contain no immunoreactivity for PrRP in any of the three brain regions (DMH, NTS, or VLM) but that PrRP immunoreactivity is rescued in the crossed *nestin*-Cre::LSL-*PrRP* mice (Figures S5 and S5B). Furthermore, relative quantitative PCR was used to demonstrate selective reactivation of the *PrRP* gene in the three brain regions of *nestin*-Cre::LSL-*PrRP* mice, but not in either the pituitary or adrenal glands (Figure S5B). Next, we crossed LSL-*PrRP* mice with *TH*-Cre to reactivate the *PrRP* gene in TH-positive cells in the NTS and VLM of the brainstem, but not in the DMH. Selective rescue of PrRP only in the brainstem (not in the hypothalamus, pituitary, or adrenal) was confirmed with immunohistochemistry and PCR (Figure S5).

**Figure 6:**
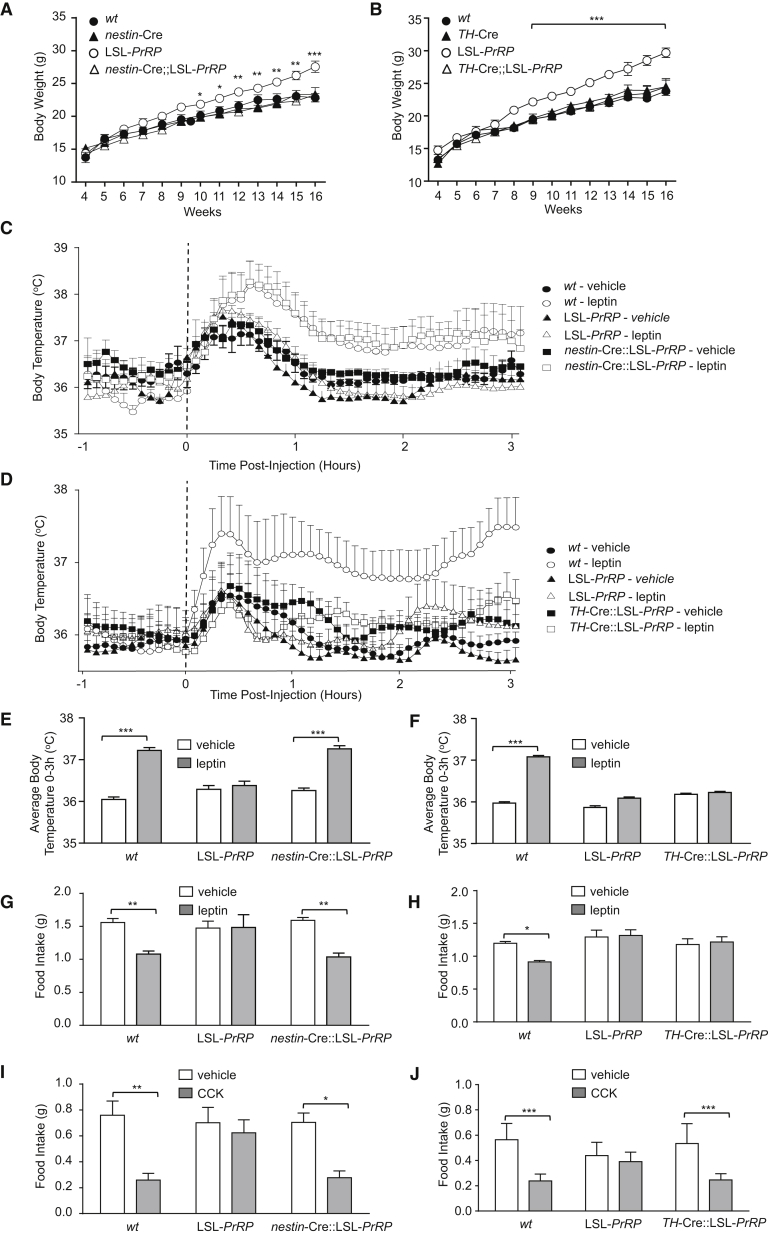
PrRP Neurons in the Hypothalamus Mediate the Thermogenic Action of Leptin, while PrRP Neurons in the Brainstem Mediate the Anorectic Action of CCK (A) Growth curves of *wt*, *nestin*-Cre, LSL-*PrRP*, and *nestin*-Cre::LSL-*PrRP* littermates. LSL-*PrRP*, which lack universal expression of *PrRP*, are obese, whereas *nestin*-Cre::LSL-*PrRP* mice, which have PrRP rescued in the brain, are not. (B) Growth curves of *wt*, *TH*-Cre, LSL-*PrRP*, and *TH*-Cre::LSL-*PrRP* littermates. *TH*-Cre::LSL-*PrRP* mice, which have *PrRP* rescued only in neurons of the NTS and VLM, do not become obese. (n = 5 to 6 per group, bars represent mean ± SEM; two-way ANOVA repeated-measures, ^∗^p < 0.05; ^∗∗∗^p < 0.001). (For validation of the rescue of PrRP expression in the two Cre crosses, see Figure S5). (C and E) Leptin administration (5 mg/kg, i.p.) acutely increases body temperature in *wt* and mice expressing *PrRP* in the brain (*nestin*-Cre::LSL-*PrRP*) (n = 6–8 per group, bars represent mean ± SEM; two-way ANOVA, ^∗∗∗^p < 0.001). (C, D, and F) Leptin administration (5 mg/kg, i.p.) acutely increases body temperature in *wt* but not mice expressing *PrRP* selectively in the brainstem (*TH*-Cre::LSL-*PrRP*) (n = 6 to 7 per group, bars represent mean ± SEM; two-way ANOVA, ^∗∗∗^p < 0.001). Dotted line in (C) and (D) represents time of injection. (G and H) The anorectic action of leptin (2 hr food intake) (G) in mice expressing *PrRP* in the brain (*nestin*-Cre::LSL-*PrRP*), and (H) in mice expressing *PrRP* in brainstem neurons only (*TH*-Cre::LSL-*PrRP*). (5 to 6 weeks old, n = 5 to 6 per group, bars represent mean ± SEM; two-way ANOVA, ^∗^p < 0.05, ^∗∗^p < 0.01). (I and J) The anorectic actions of CCK (1 hr food intake) (I) in mice expressing *PrRP* in the brain (*nestin*-Cre::LSL-*PrRP*), and (J) in mice expressing *PrRP* in brainstem neurons only (*TH*-Cre::LSL-*PrRP*). (5 to 6 weeks old, n = 5 to 6 per group; bars represent mean ± SEM; two-way ANOVA, ^∗^p < 0.05; ^∗∗^p < 0.01; ^∗∗∗^p < 0.001.)

One caveat with these studies is that the Cre lines might have a nonselective metabolic phenotype themselves and, therefore, we were extremely careful to make sure that Cre-expressing crosses were phenotyped using the relevant littermate controls (i.e., homozygous wild-type, homozygous LSL-*PrRP*, and/or heterozygous *nestin*-Cre/*TH*-Cre). Both the *nestin*-Cre and *TH*-Cre mice had the same body weight curves as their respective wild-type littermates. In both crosses, the homozygous LSL-*PrRP* littermates show divergent body weights at 8 to 9 weeks of age on normal chow when compared with the wild-type and the Cre-expressing mice; however, the obesity is fully reversed in the *nestin*-Cre::LSL-*PrRP* and *TH*-Cre::LSL-*PrRP* mice (Figures 6A and 6B). As previously noted, the obese phenotype of LSL-*PrRP* mice appears to be dependent on increased food intake, and this is reversed when PrRP expression is rescued in the brain (24 hr food intake in wild-type, *nestin*-Cre, LSL-PrRP, and *nestin*-Cre::LSL-PrRP littermates was as follows: 5.3 ± 0.1, 5.4 ± 0.1; 5.8 ± 0.1^∗^, and 5.3 ± 0.1g; ^∗^p < 0.05 LSL-PrRP versus all other groups). Preobese LSL-*PrRP* mice show neither the thermogenic response to leptin nor the anorectic response to either leptin or CCK (Figures 6C–6J), thus confirming results from our earlier experiment. However, all responses to either leptin or CCK are rescued in the *nestin*-Cre::LSL-*PrRP* littermates, highlighting the importance of brain-expressed PrRP, rather than that produced in peripheral tissues, in mediating the metabolic actions of the two hormones. As predicted, reactivation of only brainstem expression in the *TH*-Cre::LSL-*PrRP* mice rescues the response of these mice to the satiating effects of CCK (Figure 6J) and reduces hyperphagia (24 hr food intake in wild-type, *TH*-Cre, LSL-PrRP and *TH*-Cre::LSL-PrRP littermates was as follows: 5.1 ± 0.1, 5.2 ± 0.1; 6.6 ± 0.1^∗∗∗^, and 5.2 ± 0.1 g; ^∗∗∗^p < 0.001 LSL-PrRP versus all other groups). However, neither the thermogenic or energy intake actions of leptin are rescued in *TH*-Cre::LSL-*PrRP* mice (Figures 6F and 6H).

## Discussion

Leptin acts on a distributed network of neurons to affect the key determinants of body weight balance: energy intake and energy expenditure (Myers et al., 2009). Genetic isolation of phenotypically identified cell populations has described some first-order, leptin-responsive peptidergic neurons, notably those residing in the ventromedial region of the hypothalamus, that contain POMC, NPY/Agrp, and SF-1/PACAP (Balthasar et al., 2004, Dhillon et al., 2006, Hawke et al., 2009, van de Wall et al., 2007). POMC and NPY/Agrp neurons engage downstream targets, such as in the paraventricular hypothalamic nucleus, that possess the melanocortin receptor (MC4R). However, this pathway is dissociated from that which mediates the actions of leptin on energy expenditure (Balthasar et al., 2005, Haynes et al., 1999). This distinct action of leptin is achieved by activating sympathetic output from the brain to increase nonshivering, adaptive thermogenesis by brown adipose tissue (Cannon and Nedergaard, 2004, Scarpace and Matheny, 1998, Trayhurn et al., 1977). Though the neurons in the ventromedial region of the hypothalamus have been partly implicated in adaptive thermogenesis (Kim et al., 2011, Kong et al., 2012, Shi et al., 2013), compelling evidence is available for an important direct action of leptin on neurons of the dorsomedial region of the hypothalamus (Enriori et al., 2011, Zhang et al., 2011). Neurons in the DMH are part of the central circuitry that regulates brown-adipose-dependent thermogenesis in response to both leptin and cold stimulation (Enriori et al., 2011, Morrison et al., 2008, Zhang et al., 2011), but until now, their identity has remained undetermined. Here, we have defined a single population of first-order neuron that is critical for leptin’s actions on adaptive thermogenesis. PrRP neurons in the DMH are sensitive to energy status, possess Lepr, and respond to stimulation by leptin. As all of leptin’s effects on metabolism are mediated by brain-expressed Lepr (de Luca et al., 2005), and because the population in the DMH are the only PrRP neurons in the mouse to have Lepr, we can conclude that the obese phenotype of *PrRP*-Cre::*Lepr*^flox/flox^ mice is probably due to loss of function in these cells. However, bearing in mind confounders due to possible developmental expression of PrRP in nonfated PrRP neurons, future studies should consider intervention in adults (for example, by knock-down of PrRP or selective optico-/pharmaco-genetic regulation of the neurons).

We and others have previously suggested an important role for PrRP in appetite and body weight regulation and have postulated that it mediates some of the effects of leptin (Bechtold and Luckman, 2006, Dodd and Luckman, 2013, Ellacott et al., 2002, Lawrence et al., 2000, Takayanagi et al., 2008). As well as reducing food intake, central injection of PrRP causes a robust increase in core body temperature and energy expenditure (Ellacott et al., 2002, Lawrence et al., 2000, Lawrence et al., 2004). The genetic knock-out of either PrRP or its cognate receptor, GPR10, in mice produces an obese phenotype (Gu et al., 2004, Takayanagi et al., 2008). Interestingly, a natural mutation of GPR10 also occurs in the Otsuka Long-Evans Tokushima Fatty (OLETF) rat strain, which is commonly studied as it also has a mutation in the CCK_1_ receptor (Funakoshi et al., 1995). However, the obesity and diabetes of the OLETF rat is completely reversed in congenic rats with the wild-type GPR10 allele, and so caution should be applied in using this animal as a selective CCK_1_ receptor mutant (Watanabe et al., 2005). Here, we reiterated the obese phenotype in our LSL-*PrRP* mouse but then reversed the phenotype by reinstating expression not only in the brains of *nestin*-Cre::LSL-*PrRP* mice but also in brainstem selective *TH*-Cre::LSL-*PrRP* mice. These results first demonstrate that there is not complete redundancy in leptin-sensitive metabolic pathways in the brain. Second, even though brainstem PrRP neurons are involved in satiety, but are not regulated directly by leptin, there are powerful links between forebrain and brainstem circuits that act in concert to regulate body weight (McMinn et al., 2000, Myers et al., 2009, Yang et al., 2009). It is likely that, due to the integrated nature of central circuits involved in regulating metabolism, modifying a single element may have relatively strong overall effects. It is noteworthy that manipulating leptin signaling in PrRP DMH can slightly attenuate the anorexic response to leptin, even though the major influence of leptin on food intake is probably at the level of the ventromedial hypothalamus (Balthasar et al., 2005, van de Wall et al., 2007).

The DMH is an integral part of the circuitry regulating body temperature, receiving input from peripheral and central sensors, and providing output to presympathetic neurons in the midbrain and spinal cord that innervate heat-producing brown adipose tissue (Morrison et al., 2008). As well as initiating adaptive thermogenesis in response to cold stimulation, the same circuitry is engaged at the level of the DMH in response to obesogenic diets—an effect that is driven by leptin produced from white adipose tissue (Cannon and Nedergaard, 2004, Enriori et al., 2011, Zhang et al., 2011). NPY neurons in the DMH have been implicated in adaptive thermogenesis (Chao et al., 2011, Lee et al., 2013), though unlike arcuate NPY neurons, they do not contain leptin receptors and do not respond to leptin with an increase in pSTAT3 (Bi et al., 2003, Draper et al., 2010). The knock-down of NPY in the DMH by adeno-associated virus-delivered RNAi enhances the thermogenic capacity of brown adipose tissues (Chao et al., 2011), which suggests that it could have an opposing function compared with DMH PrRP. It would be interesting to hypothesize a “yin-yang” relationship between NPY and PrRP neurons in the DMH, similar to that seen with NPY and POMC neurons in the arcuate nucleus. Leptin and other metabolic signals may affect DMH NPY neurons indirectly via PrRP neurons, which together generate thermogenic output to presympathetic neurons. Indeed, the increased expression of DMH *NPY* mRNA noted in obese OLETF rats (Bi et al., 2001) might be a direct consequence of a lack of PrRP-GPR10 signaling in this model (Watanabe et al., 2005). In the current studies, we have also confirmed the role of brainstem PrRP neurons in mediating the effects of CCK, a satiety hormone released in the gut that stimulates afferent vagal nerves terminating in the NTS (Bechtold and Luckman, 2006, Lawrence et al., 2002). PrRP may reduce food intake locally by modulating vago-vagal gut reflexes, but PrRP neurons also project to upstream targets in the hypothalamus (Dodd and Luckman, 2013).

RFamides have evolutionarily conserved functions in feeding behavior and in energy balance, but their role in mammalian systems has received relatively little attention (Bechtold and Luckman, 2007). Populations of PrRP neuron in the hypothalamus and brainstem have distinct, nonredundant functions in both arms of body weight regulation: mediating the thermogenic actions of leptin and the satiating actions of CCK, respectively. Thus, they can be added as unique pieces to the distributed brain network affecting whole-body energy homeostasis.

## Experimental Procedures

### Animals

All animal procedures were carried out in accordance with the Animals (Scientific Procedures) Act 1986 (UK) and approved by the University of Manchester Animal Welfare and Ethics Review Board. Mice had access to normal chow (Beekay International, Hull) and water ad libitum unless stated otherwise. For some phenotyping studies, mice were fed on a high-energy diet (5.16 kcal/g, 18.3% protein, 60.9% fat by energy; Research Diets, New Brunswick). All mouse colonies were group housed in a temperature- (22°C ± 1°C) and humidity-controlled (45% ± 10%) environment on a 12:12 hr light/dark cycle. Experimental animals were singly housed for procedures requiring individual measurements of food intake or energy expenditure.

### Transgenic Mice

BAC genomic library clones (rTgV; GenOway, Lyon) were used for the generation of targeting construct to generate heterozygous PrRP-*Cre* and LSL-*PrRP* mice. Briefly, the rTgV BAC clone collection, containing genomic fragments of 15–25 kb in size, was screened by PCR using two *PrRP* primer pairs. The first primer pair amplifies a genomic fragment of *PrRP* (official gene name *Prlh*; accession number NM_001101647), enabling screening for the presence of the genomic fragment corresponding to the small homology arm used for targeting vector construction. The second primer pair amplifies a genomic fragment of the *PrRP* gene enabling screening for the presence of the genomic fragment corresponding to the long homology arms, which were cloned into a targeting vector. The *PrRP*-Cre targeting vector contained an IRES-Cre transgene inserted just downstream of the endogenous STOP codon in *PrRP* exon 2. The LSL-*PrRP* targeting construct incorporated *lox*P sites flanking a transcription STOP cassette just upstream the endogenous *PrRP* gene and a neomycin-resistance cassette (Figure 4A). The linearized targeting construct was transfected into embryonic stem cells, and correctly targeted clones were injected into blastocysts. High-percentage male chimeras (chimerism rate >50%) were mated with wild-type C57BL/6J mice to produce heterozygous offspring. F1 mice identified by PCR were further verified by Southern blot analysis. Heterozygous LSL-*PrRP* mice were mated with *nestin*-Cre (B6.Cg-Tg(Nes-cre)1Kln/J, C57BL/6, The Jackson Laboratory, Maine) and *TH*-Cre (B6.Cg-Tg(Th-cre)1Tmd/J, C57BL/6, The Jackson Laboratory) mice for the conditional rescue of *PrRP* in brain cells and TH-expressing cells, respectively. Immunohistochemistry and relative quantitative RT-PCR analysis confirmed the selective rescue of *PrRP* in the Cre-mouse crosses (Figure S5).

The generation of the other transgenic mice has been described elsewhere. Heterozygous *PrRP*-Cre mice were also mated with Lepr^flox/flox^ mice. This allowed for the conditional excision of leptin receptors in cells expressing Cre (Balthasar et al., 2004). The *Lepr*-Cre*::eGFP* mice are homozygous for *Lepr*-IRES-Cre and the reporter gene, Rosa^eGFP^ (Leshan et al., 2009).

### Laser-Capture Microdissection

Male, outbred CD1 mice (8 weeks old; Charles River, Sandwich) were subjected either to a 24 hr fast or 8 weeks feeding on the high-energy diet. At the end of the experiments, mice were sacrificed, and the whole brain was isolated and frozen. Fifteen micrometer coronal sections were cut by cryostat and mounted on sterile RNase-free, membrane-coated glass slides (PALM Membrane Slides; PALM Microlaser Technologies, Bernried). Each slide was immediately placed on dry ice. Within 24 hr of sectioning, the frozen sections were thawed and fixed for 30 s in 95% ethanol. All solutions were prepared with RNase-free water. Laser-capture microdissection was performed using a PALM MicrolaserSystem (PALM Microlaser Technologies) (Jovanovic et al., 2010). The DMH was microdissected from sections −1.82 to −2.20 mm from bregma and the NTS/VLM from −7.40 to −8.00 from bregma (Paxinos and Franklin, 2001). Following microdissection, the captured samples were stored at −80°C prior to RNA isolation.

### Relative Quantitative Real-Time PCR

RNA was extracted using Trizol reagent (Life Technologies, Paisley), and total RNA quality and quantity determined using a NanoDrop 3300 (Thermo Scientific, Wilmington). mRNA were reverse transcribed using a High-Capacity cDNA Reverse Transcription Kit (Applied Biosystems, Warrington) and processed for quantitative real-time PCR using the QuantiFast SYBR Green PCR Kit (QIAGEN, Manchester); β-actin was used for normalization. Relative quantification was achieved using the ΔΔ^Ct^ method. The reactions were performed in an ABI PRISM 7300 Sequence Detection System (Applied Biosystems, Warrington).

#### Primers Used in Genotyping and Real-Time Quantitative PCR

Genotyping; *LSL-PrRP floxed allele:* (F) 5′-CAC GCA CCA CAC ACA CAC GTA CAT C-3′, (F) 5′-GGA AAC AGG ACC ATT CTG GGG AGA TC-3′, (R) 5′-TTG AAT GGA AGG ATT GGA GCT ACG G-3′; *Cre allele:* (F) 5′-GCC CTG GAA GGG ATT TTT GAA GCA-3′, (R) 5′-ATG GCT AAT CGC CAT CTT CCA GCA-3′; *Lepr floxed allele*: (F) 5′-AAT GAA AAA GTT GTT TTG GGA CGA-3′, (R) 5′-CAG GCT TGA GAA CAT GAA CAC AAC AAC-3′; *eGFP:* (F) 5′-AAG TTC ATC TGC ACC ACC G-3′, (R) 5′-TCC TTG AAG AAG ATG GTG CG-3′.

Relative quantitative PCR; *PrRP:* (F) 5′-TGC TGC TGC TAG GCT TAG TC-3′, (R) 5′-CGT GTA CCA GGC AGG ATT GA-3′; *Ucp-1*: (F) 5′-ACT GCC ACA CCT CCA GTC ATT-3′. (R) 5′-CTT TGC CTC ACT CAG GAT TGG-3′; *β-actin:* (F) 5′- AGA GGG AAA TCG TGC GTG AC-3′, (R) 5′- CAA TAG TGA TGA CCT GGC CGT-3′.

### Feeding Experiments

Mice were assigned randomly to receive i.p. injection of vehicle (0.9% w/v NaCl), CCK-8 sulphated (20 μg/kg; Tocris Bioscience, Bristol), or recombinant murine leptin (5 mg/kg; Peprotech, London). Injections were made in a volume of 4 ml/kg body weight. Injections were made at lights off (2,000 hr; ZT12), and food intake was determined 1, 2, 4, and 24 hr after injection.

### Indirect Calorimetry and Body-Temperature Measurements

Indirect calorimetry cages (Columbus Instruments, Columbus) were used to measure metabolic gases (O_2_ and CO_2_) in transgenic crosses. Additional mice were surgically implanted into the peritoneal cavity, under general anesthesia, with remote telemetry devices (DataScience International, Minneapolis) 7 days prior to experiments. Mice were randomly assigned to receive vehicle (0.9% NaCl, i.p.) or recombinant murine leptin (5 mg/kg, i.p.) in a volume of 4 ml/kg during lights on (1200 hr; ZT4). Core body temperature was measured remotely 24 hr before and after injection at 5 min intervals. Data were averaged and smoothed to within ±5 min.

### Immunohistochemistry

Animals were anesthetized with isoflurane and perfused transcardially with phosphate-buffered saline containing heparin, followed by 4% paraformaldehyde in the same buffer. Brains were post fixed in 4% paraformaldehyde overnight, then placed in sucrose and cut at 30 μm on a microtome. Immunohistochemistry was performed as described (Dodd et al., 2013, Ernst et al., 2009, Loh et al., 2011). For detection of endogenous PrRP or TH, sections were incubated with an antibody to PrRP (1:500; H-008-52, Phoenix Pharmaceuticals, Burlingame) or TH (1:1000; AB1542, Merck Millipore, Billerica). Primary antibodies were visualized by further incubation with FITC-, Cy3-, or DyLight 405-conjugated secondary antibodies (all 1:1,000; Jackson Laboratories, California). For nonfluorescent pSTAT3 staining, brain sections were incubated sequentially with a primary antibody to pSTAT3 (1:500; AB9131, Cell Signaling Technologies, Boston), a biotinylated anti-rabbit secondary antibody (1:500; Vector Laboratories, UK), and a streptavidin-biotin complex (1:500; GE Healthcare, UK), and visualized with nickel-intensified diaminobenzidine (Vector Laboratories, UK). For the detection of pSTAT3 in PrRP-expressing neurons, immunohistochemistry for pSTAT3 was performed on coronal brain sections from 8-week-old male *PrRP*-Cre::eGFP mice fasted overnight and injected with either vehicle (0.9% NaCl, i.p.) or recombinant murine leptin (i.p. 5 mg/kg) in a volume of 4 ml/kg. Brain sections were first processed for pSTAT3 immunohistochemistry (as described above) and subsequently incubated with an eGFP antibody (1:1,000; AB13970, Abcam, Cambridge), which was visualized using a FITC-conjugated secondary antibody (1:1,000; Jackson Laboratories, California). The sections were photographed using a fluorescence microscope (Zeiss Axioskop, Carl Zeiss AG, Oberkochen) and assessed for pSTAT3/eGFP colocalization. pSTAT3 immunoreactivity was photographed under a bright field, while the eGFP fluorescent immunostain was photographed using an FITC filter. The pictures were merged using Adobe Photoshop 7.0 software, and colocalization was quantified visually. The number of pSTAT3-expressing cell nuclei was quantified only in brain regions containing eGFP expression.

### Statistical Analysis

Data are presented as means ± SEM. Statistical analyses were performed using Prism statistical package (GraphPad Software Inc, San Diego). Unpaired two-way t tests were used throughout to compare two distinct groups. When more than two groups were compared, a one-way or two-way ANOVA followed by Bonferroni’s multiple-comparison post hoc tests were used.

## Supplemental Information

Document S1. Figures S1–S5 and Table S1

Document S2. Article plus Supplemental Information
