# Supplementary material for: The Thermogenic Effect of Leptin Is Dependent on a Distinct Population of Prolactin-Releasing Peptide Neurons in the Dorsomedial Hypothalamus
Source: Cell Metab. 2014 Oct 7;20(4):639–49. doi: 10.1016/j.cmet.2014.07.022 (PMC4192552; doi:10.1016/j.cmet.2014.07.022)
Supplement: Document S1. Figures S1–S5 and Table S1 [file mmc1.pdf]

Cell Metabolism, Volume *20*

Supplemental Information

The Thermogenic Effect of Leptin Is Dependent on a Distinct Population of Prolactin-Releasing Peptide Neurons in the Dorsomedial Hypothalamus

Garron T. Dodd, Amy A. Worth, Nicolas Nunn, Aaron K. Korpal, David A. Bechtold, Margaret B. Allison, Martin G. Myers Jr., Michael A. Statnick, and Simon M. Luckman

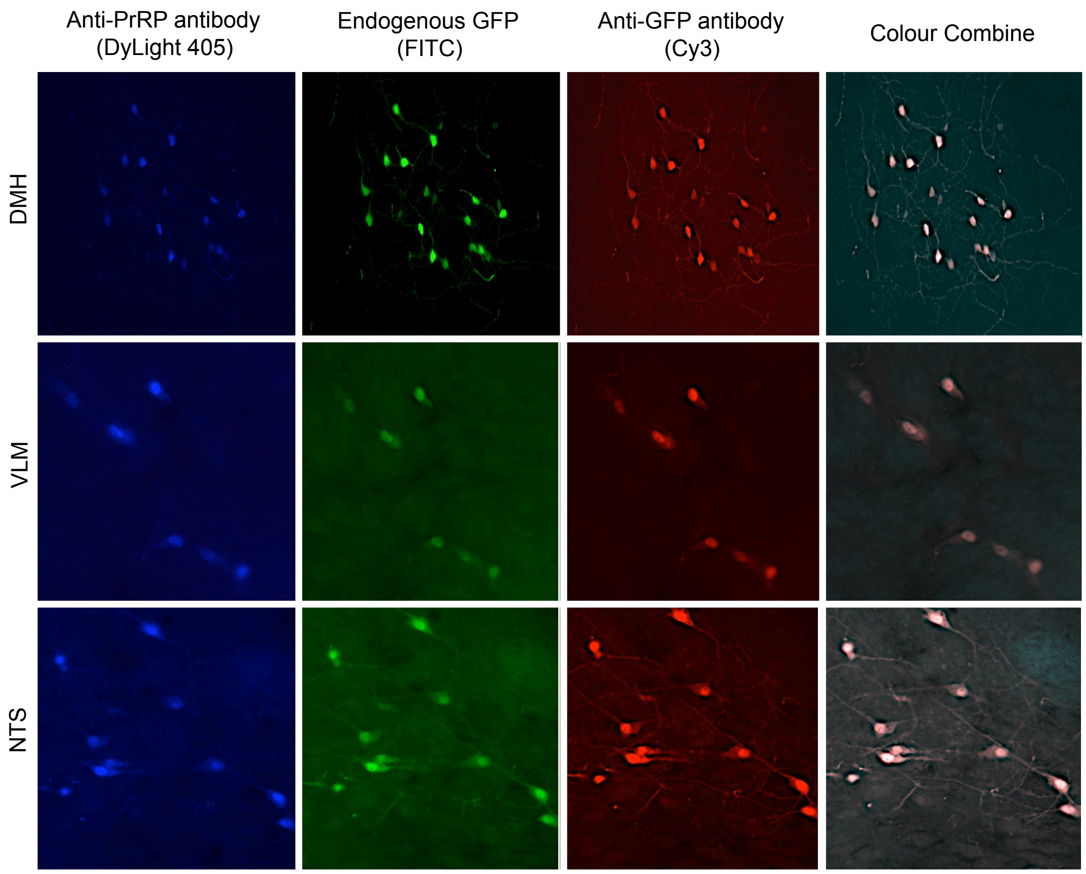

Figure S1, Related to Figure 2. Validation of the Cre recombinase mouse, demonstrating targeted recombination in PrRP neurons. Dual-label immunostaining for PrRP (blue) and eGFP (red), as well as endogenous eGFP fluorescence (green), in the DMH, NTS and VLM of *PrRP*-cre::eGFP mice to demonstrate Cre recombinase is expressed exclusively in PrRP neurones.

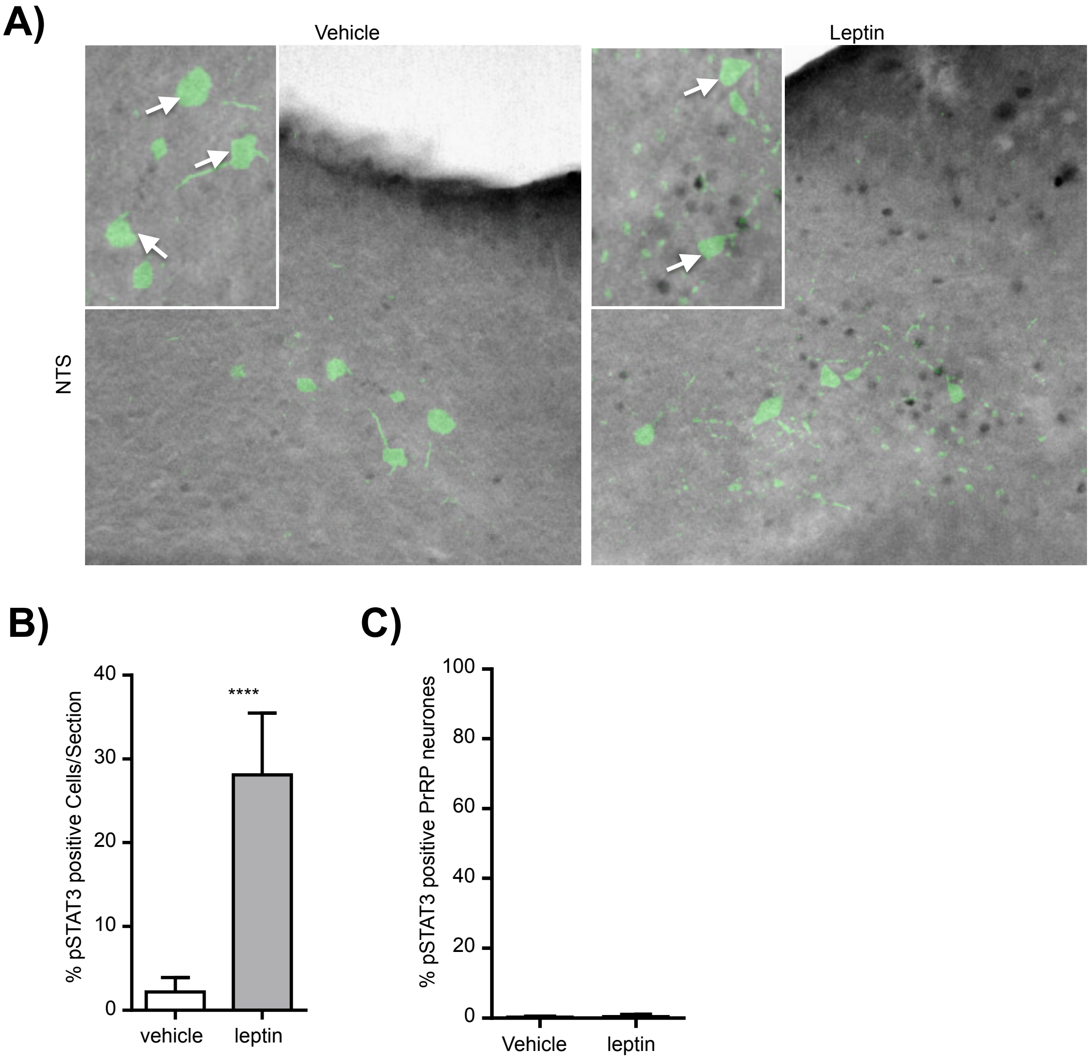

Figure S2, Related to Figure 2. PrRP neurons in the nucleus of the tractus solitarius (NTS) do not respond to leptin. A) Photomicrograph showing black-labelled nuclear pSTAT3 induction in the brainstem NTS 60min after vehicle or leptin (5 mg/kg, i.p) administration to *PrRP*-Cre::eGFP mice. PrRP neurons are immunostained for enhanced GFP. White arrows indicate single-stained PrRP neurons. Below are bar graphs showing quantification that **B)** pSTAT3 is induced in the NTS, but **C)** not in PrRP neurons (n=6 per group, bars represent mean ± SEM; unpaired t-test, ****P<0.0001). Compare with the response of PrRP neurons in the DMH, **Figure 2**.

A)

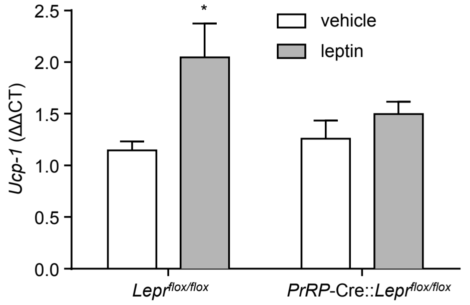

B)

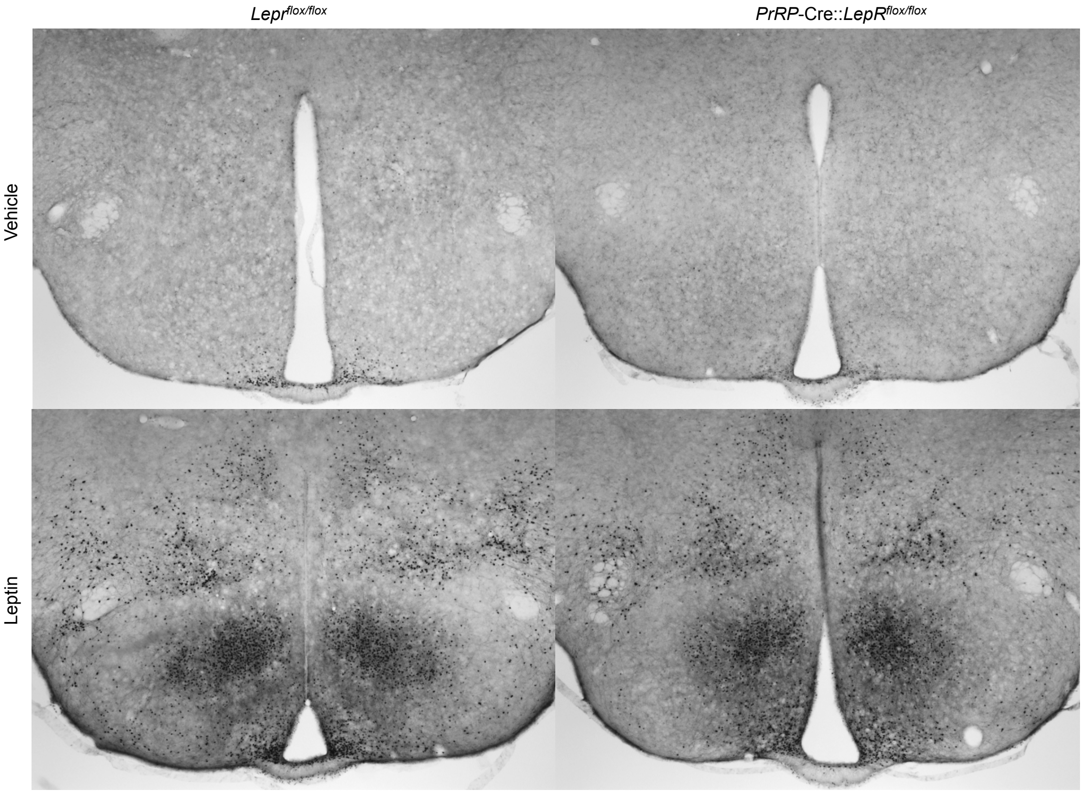

Figure S3, Related to Figure 3. The response of *PrRP*-Cre::*Lepr*$^{\mathbf{flox/flox}}$ mice to leptin. A) *PrRP*-Cre::*Lepr*$^{\text{flox/flox}}$ mice did not increase expression of *Ucp*-1 mRNA in the interscapular brown adipose depot following leptin, unlike control littermates, even though **B)** leptin caused an apparently normal pSTAT3 response in the hypothalamus.

A)

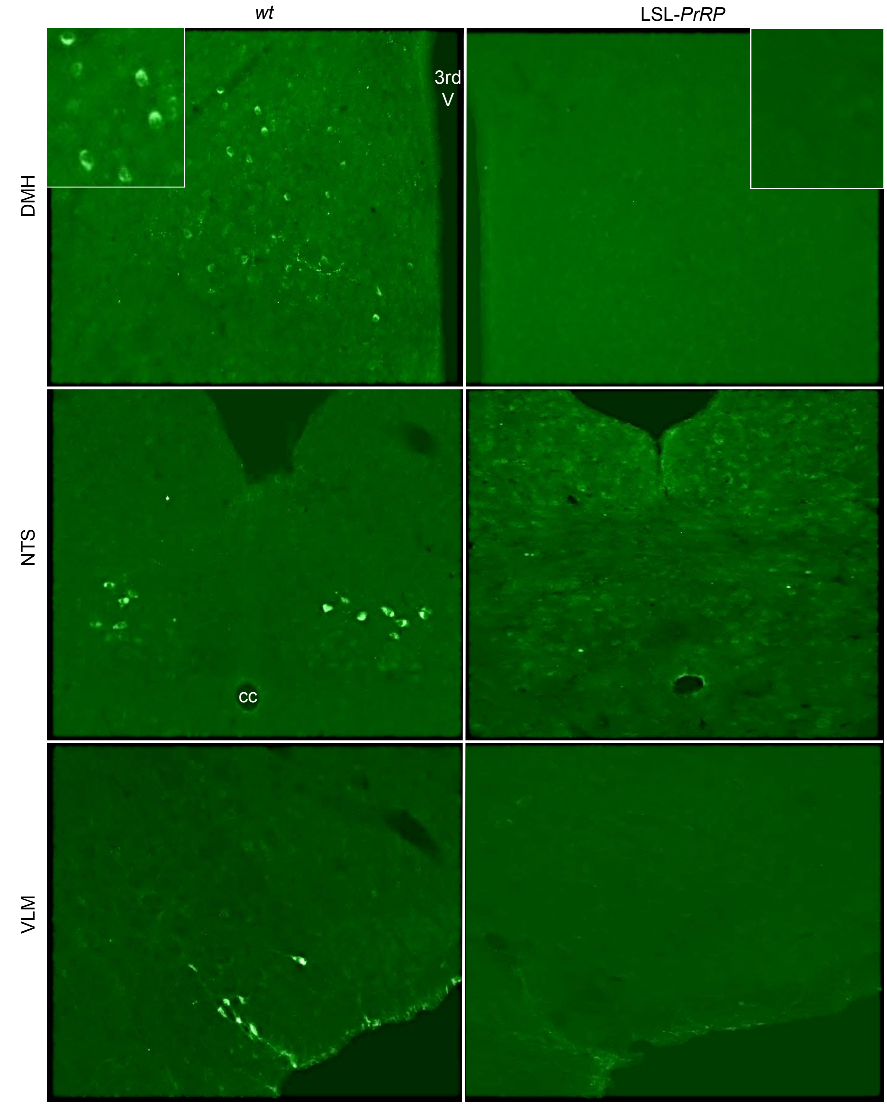

B)

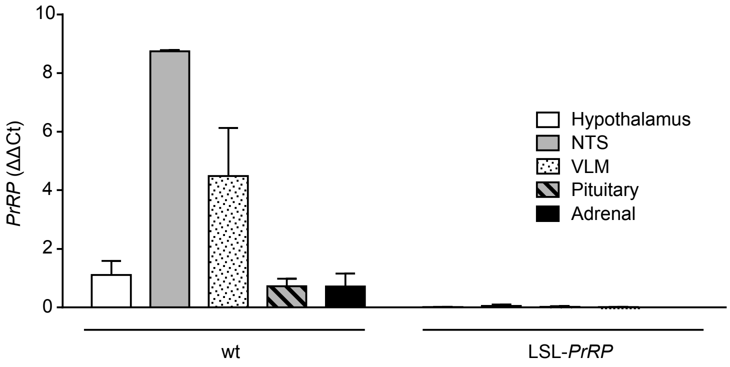

Figure S4, Related to Figure 4. Characterisation of central and peripheral PrRP expression in wild-type (*wt*) and LSL-*PrRP* mice. A) Immunofluorescent staining for endogenous PrRP protein, with representative images from the DMH, NTS and VLM. 3V = third ventricle; cc = central canal. **B)** Quantitative PCR for *PrRP* mRNA in central (DMH, NTS, VLM) and peripheral (pituitary, adrenal) tissues (n=6 animals; expressed relative to level in *wt* DMH, bars represent mean ± SEM). LSL-*PrRP* mice show no central or peripheral expression of PrRP. LSL-PrRP mice are obese when compared with their wild-type littermates (see **Figure 4**).

A)

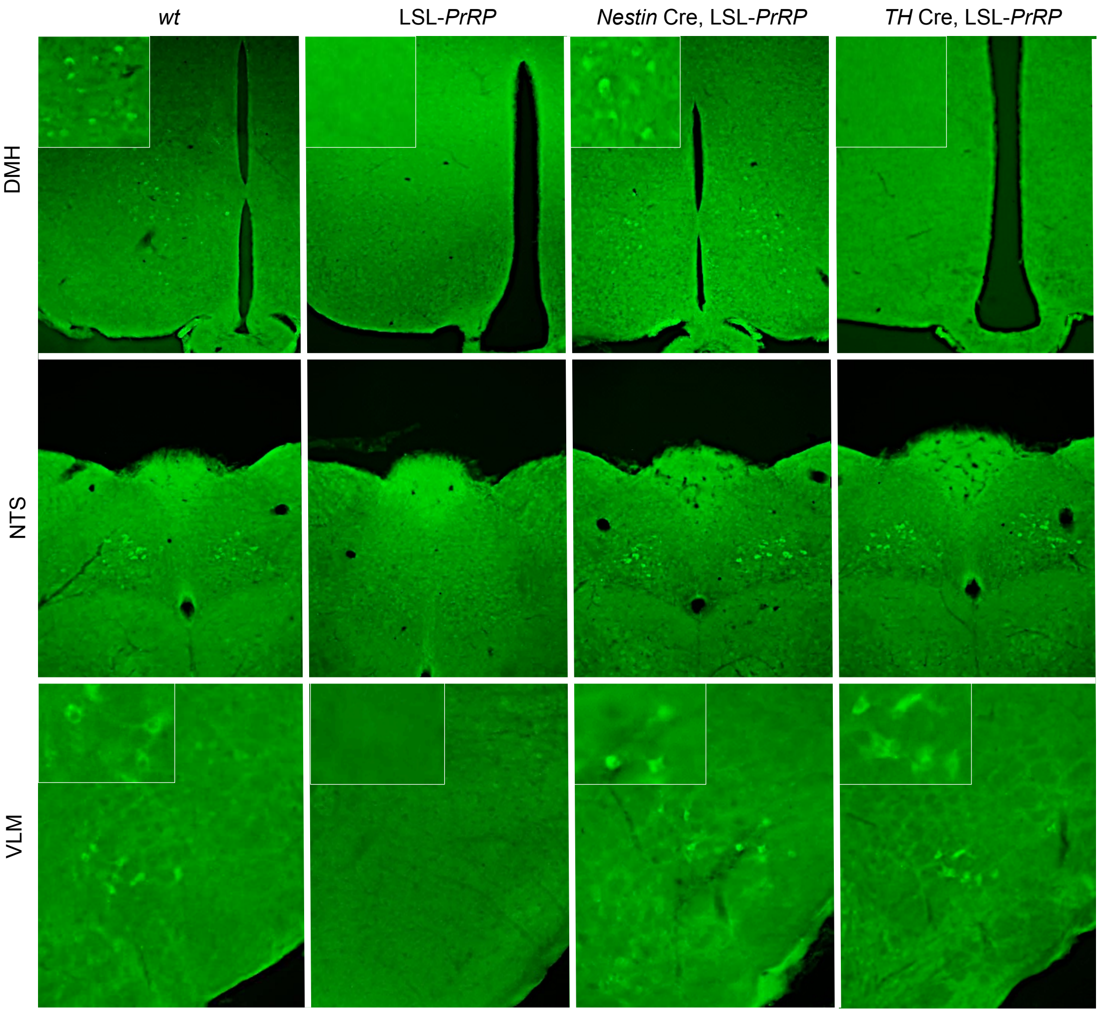

B)

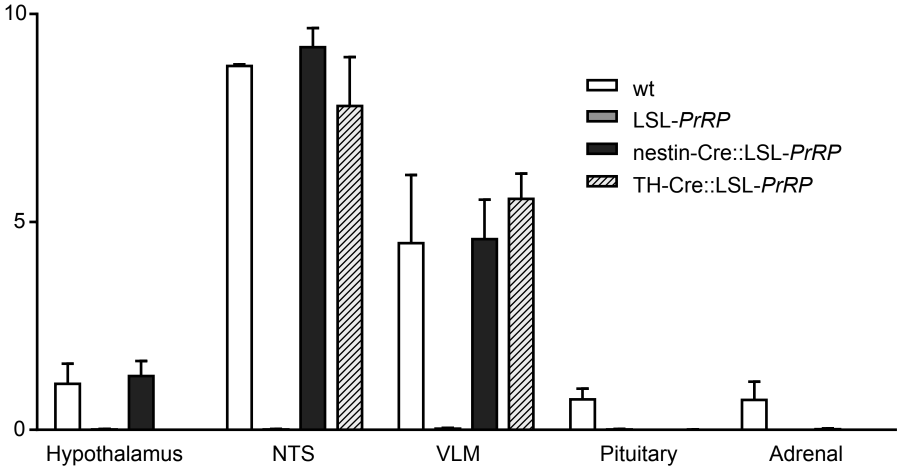

Figure S5, Related to Figure 6. Characterisation of PrRP expression in wild-type (*wt*), LSL-*PrRP*, *nestin*-Cre::LSL-*PrRP* and *TH*-Cre::LSL-*PrRP* mice. A) Immunofluorescent staining for endogenous PrRP protein, with representative images from the DMH, NTS and VLM. 3V = third ventricle; cc = central canal. **B)** Quantitative PCR for *PrRP* mRNA in central (DMH, NTS, VLM) and peripheral (pituitary, adrenal) tissues (n=6 animals; expressed relative to level in *wt* DMH, bars represent mean ± SEM). N*estin*-Cre::LSL-*PrRP* mice show complete rescue of PrRP expression in the brain, but not peripheral tissues. *TH*-Cre::LSL-*PrRP* mice show exclusive rescue of PrRP expression in the brainstem (NTS and VLM) only. Comparisons made between littermates and functional rescue are demonstrated in **Figure 6**.

Supplementary Table S1	***Lepr*$^{flox/flox}$**	***PrRP*-Cre:: *Lepr*$^{flox/flox}$**	***wt***	**LSL-*PrRP***
**Age at puberty (vaginal opening; day)**	**41.0±2.4**	**40.6±4.6**	**37.8±1.5**	**40.8±1.4**
**O_2 consumption (8-10 weeks; ml/kg/h)**	**3069±31**	**2898±56 ***	**2833±76**	**2706±89**
**CO_2 production (8-10 weeks; ml/kg/h)**	**3102±55**	**3045±85**	**2753±87**	**2689±101**
**Respiratory Exchange Ratio**	**1.01±0.01**	**1.04±0.02**	**0.97±0.01**	**0.99±0.01**
**Average body temperature (^{o}C)**	**36.83±0.08**	**36.57±0.097 ***	**36.39±0.096**	**35.98±0.101$^{\#\#}$**
**24-h food intake (age 7 weeks; g)**	**5.16±0.196**	**5.00±0.195**	**3.66±0.138**	**3.78±0.266**
**24-h food intake (age 10 weeks; g)**	**6.25±0.300**	**6.12±0.297**	**4.63±0.306**	**4.99±0.314**
**24-h food intake (age 13 weeks; g)**	**6.70±0.285**	**6.93±0.105**	**5.16±0.129**	**5.65±0.133$^{\#}$**
**24-h food intake (age 16 weeks; g)**	**7.48±0.079**	**7.53±0.172**	**5.05±0.118**	**6.31± 0.167$^{\#\#\#}$**
**Blood glucose (fed) (age 6-8 weeks;mmol/l)**	**10.62±0.204**	**10.28±0.242**	**10.93±0.216**	**10.55±0.503**
**Blood glucose (fasted) (age 6-8 weeks;mmol/l)**	**8.38±0.153**	**8.23±0.320**	**8.06±0.149**	**8.38±0.205**
**Blood glucose (fed) (age 16 weeks; mmol/l)**	**11.83±0.308**	**11.15±0.404**	**10.17±0.138**	**11.68±0.215*****
**Blood glucose (fasted) (age 16 weeks; mmol/l)**	**8.75±0.226**	**8.18±0.149**	**7.711±0.188**	**8.78±0.188****
**Plasma leptin (age 6 weeks; ng/ml)**	**4.68±0.315**	**4.26±0.602**	**4.08±0.267**	**4.40±0.380**
**Plasma leptin (age 16 weeks; ng/ml)**	**5.10±0.171**	**6.58±0.470***	**4.83±0.401**	**5.83±0.581$^{\#\#}$**
**Plasma insulin (age 16 weeks; ng/ml)**	**1.54±0.432**	**3.61±0.768***	**1.35±0.326**	**4.70±0.856$^{\#}$**
**Epididymal fat mass (age 16 weeks; g)**	**0.78±0.042**	**1.05±0.057****	**0.615±0.057**	**1.15±0.095$^{\#\#\#}$**
**Subcutaneous fat mass (age 16 weeks; g)**	**0.33±0.0247**	**0.58±0.094****	**0.22±0.335**	**0.42±0.033$^{\#\#}$**
**Interscapular brown fat mass (age 16 weeks; g)**	**0.19±0.015**	**0.15±0.018**	**0.17±0.016**	**0.21±0.028**
**Body length (nose to tail base; cm)**	**15.73±0.269**	**15.77±0.196**	**13.52±0.212**	**13.57±0.1836**

Table S1, Related to Figures 3 and 4. Additional phenotypic characterisation of *PrRP*-Cre::*Lepr*$^{flox/flox}$ and LSL-*PrRP* mice. Values are mean ± SEM; unpaired t-test; *P<0.05, **P<0.01, ***P<0.001 compared with *Lepr*$^{flox/flox}$ littermates; $^{\#}$P<0.05, $^{\#\#}$P<0.01, $^{\#\#\#}$P<0.001 compared with wild-type littermates. The growth curves for these mice are represented in **Figures 3 and 4**.
